# Neural and sympathetic activity associated with exploration in decision-making: further evidence for involvement of insula

**DOI:** 10.3389/fnbeh.2014.00381

**Published:** 2014-11-10

**Authors:** Hideki Ohira, Naho Ichikawa, Kenta Kimura, Seisuke Fukuyama, Jun Shinoda, Jitsuhiro Yamada

**Affiliations:** ^1^Department of Psychology, Nagoya UniversityNagoya, Japan; ^2^Department of Psychiatry and Neurosciences, Hiroshima UniversityHiroshima, Japan; ^3^Human Technology Research Institute, National Institute of Advanced Industrial Science and TechnologyTsukuba, Japan; ^4^Chubu Ryogo Center, Kizawa Memorial HospitalMinokamo, Japan

**Keywords:** decision-making, exploration, entropy, positron emission tomography (PET), sympathetic activity

## Abstract

We previously reported that sympathetic activity was associated with exploration in decision-making indexed by entropy, which is a concept in information theory and indexes randomness of choices or the degree of deviation from sticking to recent experiences of gains and losses, and that activation of the anterior insula mediated this association. The current study aims to replicate and to expand these findings in a situation where contingency between options and outcomes is manipulated. Sixteen participants performed a stochastic decision-making task in which we manipulated a condition with low uncertainty of gain/loss (contingent-reward condition) and a condition with high uncertainty of gain/loss (random-reward condition). Regional cerebral blood flow was measured by ^15^O-water positron emission tomography (PET), and cardiovascular parameters and catecholamine in the peripheral blood were measured, during the task. In the contingent-reward condition, norepinephrine as an index of sympathetic activity was positively correlated with entropy indicating exploration in decision-making. Norepinephrine was negatively correlated with neural activity in the right posterior insula, rostral anterior cingulate cortex, and dorsal pons, suggesting neural bases for detecting changes of bodily states. Furthermore, right anterior insular activity was negatively correlated with entropy, suggesting influences on exploration in decision-making. By contrast, in the random-reward condition, entropy correlated with activity in the dorsolateral prefrontal and parietal cortices but not with sympathetic activity. These findings suggest that influences of sympathetic activity on exploration in decision-making and its underlying neural mechanisms might be dependent on the degree of uncertainty of situations.

## Introduction

Electrophysiological (Denburg et al., 2006; Yen et al., 2012), pharmacological (Rogers et al., 2004), human lesion (Bechara et al., 1999; Gläscher et al., 2012) studies have verified a notion that activity of the sympathetic nervous system can affect decision-making (Bechara et al., 2000; Bechara and Damasio, 2005). The insula has been identified as a pivotal brain region for this phenomenon, because the insula receives all bodily inputs including peripheral sympathetic activity, and is thought to form an integrated representation of bodily states (Craig, 2009; Critchley, 2009). Furthermore, as the insula, especially its anterior portions, has tight connections with cognition and emotion-related brain regions such as the prefrontal cortex, amygdala, and anterior cingulate cortex (ACC) (Augustine, 1996), it has been proposed that bodily states including sympathetic activity can modulate decision-making through the mediation of changes of insular activity (Damasio, 1994).

Nevertheless, there remains a controversy about a direct role of sympathetic activity on decision-making (Dunn et al., 2006; Rolls, 2014), partly as sympathetic responses are too late to instantaneously affect decision-making (Nieuwenhuis et al., 2010). Considering kinetics of sympathetic nerves, it is reasonable to hypothesize that sympathetic activity might affect tonic states or modes of decision-making within relatively longer time-scales, rather than a specific decision at a local moment. We previously tested this possibility by examining effects of sympathetic activity on a dimension of exploitation and exploration, as an aspect of the tonic states of decision-making in stochastic reversal learning (Ohira et al., 2013). Exploitation is a strategy to stick to an option that has delivered reward at the highest possibility, and thus has the greatest utility. On the other hand, exploration is a strategy to seek for new and previously unexplored options, and thus means deviations from exploitation. While exploitation is more adaptive in a stable environment, organisms have to take the strategy of exploration in an unstable environment. In this sense, the relationship between exploitation and exploration is a trade-off and the balance between these two strategies is critical for survival of animals and humans.

On the basis of previous studies (Lee et al., 2004; Seo and Lee, 2008; Baek et al., 2013; Takahashi et al., 2013, 2014), we quantitatively represented the degree of exploration by using entropy, which is a concept in information theory (Shannon, 1948). Specifically, we calculated the conditional entropy representing the degree of dependence on the immediately previous outcomes in choices of options. Larger values of entropy mean that the strategy of decision-making is the more deviated from a fixed pattern just depending on immediately previous outcomes, and is the more exploratory. As a condition of a state to calculate entropy, an outcome in the immediately previous trial was considered. This was on the basis of a previous finding in humans that an outcome in the immediately previous trial as a history of experiences of reinforcement explained a large portion of following decision-making, while influences of outcomes in older trials decayed exponentially in a stochastic learning task (Katahira et al., 2011). Another index of exploration is probability of choice of an optimal option on the basis of expected values calculated in computational reinforcement learning models (Daw et al., 2006; Badre et al., 2012). While this parameter, which is sometimes called “inverse temperature,” is usually sensitive and can dynamically vary in a trial-by-trial manner along the progress of learning, entropy represents more tonic states of randomness of choices within relatively larger numbers of trials. Therefore, we adopted entropy as an index of exploration because we aimed to elucidate influences of sympathetic activity on tonic aspects of decision-making as described above.

Our results (Ohira et al., 2013) showed that an increase of epinephrine in the peripheral blood as an index of sympathetic activity was associated with larger values of entropy indicating greater tendency of exploration. The increase of epinephrine was positively correlated with brain activity in the right anterior insula, dorsal ACC, and dorsal pons [near the locus coeruleus (LC)]. Furthermore, activity in the anterior insula mediated this correlation between epinephrine and entropy. In this study, the association of sympathetic responses and exploration was found only after introduction of the reversal of the association between options and outcomes, but not during the initial learning stage before the reversal. This suggests that the effects of sympathetic activity were not fixed, but were tuned based on evaluation of situations. To our knowledge, this was the first report of an association between peripheral sympathetic responses and exploration in decision-making, and its underlying neural mechanisms. Apparently, further evidence is needed to support the findings.

Therefore, the present study aimed replication and expansion of our previous findings (Ohira et al., 2013), by examining whether association of neural and sympathetic activities with exploration in decision-making can be modulated by uncertainty, which is one of the important factors in decision-making. For this purpose, we report results of new analyses of an available dataset of our research project where behavioral, ^15^O-water positron emission tomography (PET), EEG, cardiovascular, neuroendocrine, and immune parameters were measured during a stochastic decision-making task. In that task, we manipulated the degree of contingency between options and outcomes (monetary gains and losses) to examine variations of association between the brain and autonomic activities during decision-making corresponding to uncertainty of situations. In a condition with lower uncertainty called the contingent-reward condition, an advantageous option, compared to a disadvantageous option, is associated with monetary gains at a higher probability and with monetary losses at a lower probability. On the other hand, in another condition with higher uncertainty called the random-reward condition, the gains and losses were delivered randomly for both stimuli. Thus, the situation was substantially stochastic and participants could not learn the contingency. One merit of utilization of this dataset is that involvement of brain regions which are well known to relate to decision-making, including the anterior cingulate, orbitofrontal, and dorsolateral cortices (ACC, OFC, and DLPFC, respectively) and dorsal striatum, during the stochastic decision-making task has been clarified and published elsewhere (Ohira et al., 2009, 2010). Compared with the contingent-reward condition, the OFC, DLPFC, and dorsal striatum were dominantly activated in the random-reward condition, where participants had to continue to seek contingency between options and outcomes.

Specifically, the novelty of the present article is to examine whether functional associations between sympathetic activity, its neural representation, and exploration in decision-making indexed by entropy varies with uncertainty in decision-making. For this aim, we analyzed a correlation matrix between exploration indexed by entropy, regional cerebral blood flow (rCBF) measured by ^15^O-water PET, catecholamine (epinephrine and norepinephrine) in peripheral blood, and cardiovascular indices (heart rate (HR), mean blood pressure (MBP), total peripheral resistance (TPR), and heart rate variability (HRV) representing vagal (parasympathetic) activity (Sayers, 1973). Because we have repeatedly reported that cardiovascular, endocrine, and immune responses are down-regulated in a highly uncertain situation of stochastic decision-making (Kimura et al., 2007; Ohira et al., 2009, 2010), we expected that the association between sympathetic activity, exploration, and underlying brain activity would be observed more dominantly in the contingent-reward condition, but such associations would be weakened in the random-reward condition.

## Methods

### Participants

Sixteen healthy right-handed Japanese male undergraduate and graduate students who had no past history of psychiatric and neurological illness were recruited (M ± SD; 21.69 ± 2.25 years). They gave written informed consent in accordance with the Declaration of Helsinki. The present study was approved by the Ethics Committee of Kizawa Memorial Hospital.

### Task and procedure

#### Stochastic decision-making task

**Figure 1:**
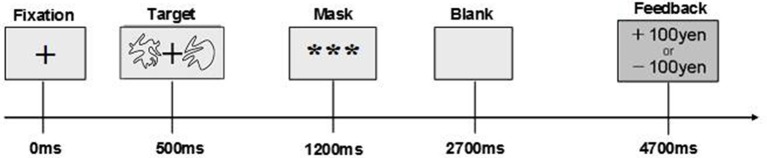
**Time course of a trial in stochastic decision-making task**.

The timeline of a trial of the stochastic decision-making task which participants performed is shown in Figure 1. Following presentation of a hair-cross as a fixation, two abstract line drawings were presented for 700 ms on the left and right side of the fixation. The drawings were selected from the set of Novel Shapes, which were validated for levels of verbalization, association, and simplicity (Endo et al., 2001). Participants chose one of the two stimuli by pressing a key within 700 ms. After that, a feedback signal indicating a gain of 100 Japanese Yen (JPY) or a loss of 100 JPY was presented. If participants did not choose a stimulus within 700 ms, they lost 100 JPY. In the contingent-reward condition, one stimulus (advantageous stimulus) led to gain at a probability of 70% but with loss at a probability of 30%, and the other stimulus (disadvantageous stimulus) was linked with gain and loss at reversed probabilities (30% reward and 70% loss). By contrast, both stimuli were linked with gain and loss at probabilities of 50%, in the random-reward condition. In this condition, the advantageous stimulus was operationally defined as a stimulus that was randomly selected by the experimenters. The verbal instruction to participants was that this task was a gamble on each trial. In addition, they were told that the amount of money that would be paid for participation in the experiment would be increased or decreased according to their performance in the task. Furthermore, we set the control condition for subtraction analyses of PET (data shown in Ohira et al., 2010). The task in the control condition was identical to that in the other two conditions, except that the computer made a decision on each trial, and participants pressed a key that the computer indicated. In all conditions, the sides of stimuli (left vs. right) were randomized, thus the task is object learning but not spatial learning. The same pair of two stimuli was presented through blocks per each condition.

#### Experimental procedure

Participants performed eight blocks of the decision-making task. Three blocks were for the contingent-reward condition, three blocks were for the random-reward condition, and two blocks were for the control condition. Each block lasted for 4 min, with an 11-min interval from the previous block, and contained 40 trials. Each condition was consisted of three continuous blocks, and the order of the contingent-reward condition and the random-reward condition was counterbalanced between the participants. Both in the contingent-reward condition and in the random-reward condition, the advantageous and disadvantageous stimuli were counter-balanced between participants, and the same stimulus was delivered as an advantageous stimulus in all blocks for a participant. Blocks for the control condition were placed in the 1st and 5th block, such that a control condition was followed by either blocks of the contingent-reward condition or blocks of the random-reward condition. The contingency between stimuli and outcomes in each control block was matched to the in the following experimental blocks; i.e., 70:30% gain/loss mapping to stimuli in one control block and 50:50% gain/loss mapping to stimuli in the other control block. Participants were told that gain and loss in the control conditions would also influence the money paid for participation.

PET scanning to collect rCBF data was conducted in each block. Cardiovascular parameters (MBP, HR, and TPR) were measured for 2 min before each block as baseline and for 4 min during the task. For measurement of plasma catecholamine (epinephrine and norepinephrine), blood samples were taken using a heparinized 22-gage butterfly catheter placed in the antecubital vein of the right forearm, for 1 min just before the baseline period of measurement of cardiovascular parameters and for the last 1 min of each block. Finally, participants were remunerated. Although participants were told that their payment would depend on their performance, all participants were paid 15,000 JPY (140 USD) for participation.

### Behavioral indices

Task performance was evaluated in two behavioral indexes: response bias and reward acquisition. Response bias means the rate of choice of the advantageous stimulus. Reward acquisition was defined as the rate of getting gain regardless of choice of advantageous or disadvantageous stimulus. Following our previous study (Ohira et al., 2013), Shannon's (1948) entropy as an index of exploration was calculated from data of participants' decisions. First, we determined a conditional probability of an action (*a*) under a state (*S*). Here, the action is a choice of the same stimulus that was chosen in the previous trial or that of another stimulus that was not chosen in the previous trial (Stay or Shift). The state is an outcome (gain or loss) in the previous trial. Thus, the conditional probability *P*(*a|S*) is calculated as follows:

$$P(a|S)=\frac{Num(a|S)+c}{\sum_{k}\left\{Num(k|S)+c\right\}},$$

where *Num*(*a|S*) is the number of Stay or Shift (*a*) under a state *S*, and *Num*(*k|S*) is the number of total choices *k* under a state *S*. The constant *c* was introduced to stabilize the calculated probability, and was fixed to 1 here. Therefore, four conditional probabilities were calculated: (1) Stay (choice of the same stimulus chosen in the previous trial) when gain was given in the previous trial, (2) Stay when loss was given in the previous trial, (3) Shift (choice of different stimulus not chosen in the previous trial) when gain was given in the previous trial, and (4) Shift when loss was given in the previous trial. Then, entropy H was estimated as follows:

$$H=-\frac{1}{N}\sum_{S}\sum_{a}P(a|S)\log_{2}P(a|S),$$

where *N* is a number of states *S*. The value of entropy *H* was standardized from 0 to 1 by dividing by *N* (here, *N* = 2). Thus, entropy calculated by this formula reflects the degree of deviation from dependence of a choice on the outcome of the previous trial. If a participant chooses the same stimulus regardless of whether it is advantageous or disadvantageous in all trials, *H* will be a minimum (approaching to 0, but *H* will not be 0 by the effect of the constant *c*). If a participant always chooses the same stimulus as the previous trial when gain was given in the previous trial and shifts the choice when loss was given in the previous trial (the Win-Stay, Lose-Shift), *H* will also be the minimum. These patterns of decision-making can be regarded as fixed strategies, independently from task performance reflected by response bias and reward acquisition. Conversely, if a participant chooses a stimulus totally independently from the outcome in the previous trial in all trials (random choice), entropy *H* will be a maximum (approaching to 1). Response bias, reward acquisition, and entropy were determined at each block of the contingent-reward and random-reward conditions, respectively.

### Autonomic indices

#### Cardiovascular responses

We recorded MBP and HR by using the finger cuff of a Portapres Model 2 (Finapres Medical Systems Inc., Amsterdam, The Netherlands) which was attached to the third finger of the dominant arm of each participant. HR was also measured and analyzed by using photoplethysmography using the Portapres at a sampling rate of 200 Hz, and the Beatfast software using a model flow. TPR was obtained by analyzing the sampled arterial pressure waveforms with the Beatfast software. Mean values of MBP, HR, and TPR were calculated for 2 min just before the task as baseline and during 4 min of the task in each block, for analyses.

We further measured components of HRV on the basis of HR data as indices of sympathetic and parasympathetic activity. Similar to other cardiovascular indexes, HRV was analyzed for 2 min just before the task for baseline and 4 min during the task in each block. First, the tachogram data on interbeat-intervals were re-sampled at 4 Hz to obtain equidistant time-series values. Then, a power spectral density was obtained by a fast Fourier transformation. The data were linearly detrended and filtered through a rectangular window. The integral of the power spectrum was measured in a low-frequency band (LF, 0.04–0.15 Hz) and a high-frequency band (HF, 0.15–0.4 Hz). Herein, we report the absolute value of HF power as an index of parasympathetic activity. For statistical analyses, we examined the LF and HF component expressed as natural logarithm values of the percentages of LF power and HF power of the total power in the spectrum (Perini et al., 2000). We then calculated the ratio of LF to HF (LF/HF), which reflects the sympatho-vagal balance (relative increase of sympathetic activity to parasympathetic activity) (Task Force of the European Society of Cardiology, The North American Society of Pacing and Electrophysiology, 1996).

#### Catecholamine

Blood samples were anticoagulated with ethylenediamine tetra-acetate, chilled, and centrifuged. Then the plasma was removed and frozen at −80°C for storage until the analysis. Epinephrine and norepinephrine in plasma were measured by using high performance liquid chromatography. Alumina was used for extraction, and the recovery rate for all amines as evaluated with a dihydroxybenzylamine standard, was between 60 and 70%. The intra-assay coefficient of variation was less than 5% for measurement of epinephrine and the inter-assay variations were less than 6% for measurement of norepinephrine.

### Statistical analyses for behavioral and autonomic indices

We performed two-way (Condition [contingent-reward vs. random-reward] × Block [1, 2, 3]) repeated-measures analyses of variance (ANOVAs) for data of response bias, reward acquisition, and entropy, separately. Three-way (Condition [contingent-reward vs. random-reward] × Period [baseline vs. task] × Block [1, 2, 3]) repeated-measures ANOVAs were performed for autonomic data (MBP, HR, TPR. epinephrine, norepinephrine, the LF/HF ratio of HRV, and the HF component of HRV). The Greenhouse-Geisser epsilon correction factor, ε (Jennings and Wood, 1976), was used where necessary. When significant interactions were found by ANOVAs, *post-hoc* analyses using Tukey's test (*p* < 0.05) were performed to detect combinations of data points which showed significant differences.

Next, to explore relational structures within the behavioral and autonomic indices, correlations within the behavioral indices (response bias, reward acquisition, and entropy) and change scores of autonomic indices (MBP, HR, TPR. epinephrine, norepinephrine, the LF/HF ratio of HRV, and the HF component of HRV) were examined. Furthermore, we performed step-wise regression analyses by using change scores of autonomic indices (MBP, HR, TPR, epinephrine, norepinephrine, the LF/HF ratio of HRV, and the HF component of HRV) as independent variables in the contingent-reward condition and random-reward condition, separately, to examine the effects of sympathetic and parasympathetic parameters on entropy. To calculate the change scores of the autonomic indices, subtractions of the autonomic indices at baseline from values during the task in each block were conducted first. Mean scores of the subtracted values within three blocks were then calculated for each indices both in the contingent-reward condition and in the random-reward condition, and used for the regression analyses.

### Neuroimaging by PET

#### Image acquisition

The distribution of rCBF was measured by using a PET scanner (General Electric Advance NXi) in a high-sensitivity three-dimensional mode at each block. A venous catheter for administering the tracer was inserted in an antecubital fossa vein in the left forearm of each participant. The participant's head was fixed in an inflatable plastic head-holder that prevented head movement. Then, a transmission scan using a rotating ^68^germanium pin source was completed for 10 min. 370-MBq bolus injection was started 60 s after initiation of each block. Scanning was started 30 s after initiation of bolus injection and continued for 60 s. The integrated radioactivity accumulated during the scanning was measured as the index of rCBF. Eight scans were performed for each participant, and the 15 min interval between successive scans was placed for clearance of radioactive levels. A Hanning filter was used to reconstruct images into 35 planes with 4.5 mm thickness and a resolution of 2 × 2 mm (full width half maximum).

#### Image processing and analyses

We used SPM 99 (Friston et al., 1995) implemented in Matlab (v. 5.3, The Mathworks Inc., Sherborn, MA, USA) for spatial preprocessing and statistical analyses of PET images. First, the images were realigned by using sinc interpolation to remove artifacts. Then, the images were transformed into a standard stereotactic space. After that, the images were corrected for whole brain global blood flow by proportional scaling and smoothed using a Gaussian kernel to a final in-plane resolution of 8 mm at full width at half maximum.

Brain activation during the contingent-reward and random-reward conditions has been previously reported (Ohira et al., 2010). Because the main interest of the current study was to examine brain regions that showed synchronized activity with autonomic activity and mediated association between the autonomic activity and exploration in decision-making, correlation maps were composed in the contingent-reward condition and in the random-reward condition, respectively. First, correlations between rCBF and the autonomic indices (MBP, HR, TPR, epinephrine, norepinephrine, the LF/HF ratio of HRV, and the HF component of HRV) that showed a significant contribution to entropy in the regression analyses described above were examined in both conditions. Change scores of the autonomic indices were used as covariates for the correlation analyses of PET images. Though whole brain activation was examined and reported (see **Tables 4, 5**) for the correlation analyses, we focused on the prefrontal, limbic, and striatum areas for interpretations, as we had found neural activity and neuro-autonomic associations in such regions during similar tasks of decision-making (Ohira et al., 2009, 2010, 2011, 2013). Next, we examined correlations between rCBF and entropy in the contingent-reward and random-reward conditions, respectively. For all correlation analyses, we adopted the statistical threshold of *p* < 0.001 (uncorrected) and *K* > 10. This threshold is relatively liberal in the current standard. However, it was chosen considering the balance between risks of the type-1 error and type-2 error (Lieberman and Cunningham, 2009) in a PET study with limited statistical power compared to fMRI studies.

## Results

### Behavioral data

Means (*Ms*) and standard errors (*SEs*) of response bias, reward acquisition, and entropy are shown in Table 1. A main effect of Condition for response bias [*F*_(1, 15)_ = 7.10, *p* < 0.05, η^2^*p* = 0.32] was significant, but neither a main effect of Block nor an interaction of Condition and Block was significant (*F* < 1.78), for response bias. Naturally, reward acquisition showed similar results as response bias, namely, a significant main effect of Condition [*F*_(1, 15)_ = 13.49, *p* < 0.01, η^2^*p*= 0.47]. No significant effect was obtained on entropy (*F* < 1.00, ns.).

**Table 1:** **Means and standard errors of behavioral indices**.

	**Contingent-reward**	**Random-reward**	**ANOVA**
Response bias (rate)	0.68 (0.06)	0.46 (0.06)	C^*^
Reward acquisition (rate)	0.57 (0.02)	0.47 (0.01)	C^**^
Entropy (bit)	0.64 (0.06)	0.74 (0.06)	ns

The column of ANOVA indicates significance of main effects of Condition (C) and Block (B) and an interaction (C × B) in analyses of variance;

* p < 0.05;

** *p < 0.01; ns, non-significant*.

Entropy was not correlated with response bias and reward acquisition in either condition [see **Table 3**; *r*_(14)_ < 0.17, ns.], indicating that entropy is independent of performance of the decision-making task.

### Autonomic data

*Ms* and *SEs* of autonomic indices in each condition are shown in Table 2. For MBP, ANOVA showed significant main effects of Condition and Period [*F*_(1, 15)_ = 6.18, *p* < 0.05, η^2^*p* = 0.29; *F*_(1, 15)_ = 37.58, *p* < 0.001, η^2^*p* = 0.71], suggesting that MBP in the contingent-reward condition was higher than that in the random-reward condition, and that MBP elevated during the task compared to the baseline. For HR, a significant main effect of Condition was shown [*F*_(1, 15)_ = 6.92, *p* < 0.05, η^2^*p* = 0.32], indicating that HR was higher in the contingent-reward condition compared with that in the random-reward condition. TPR showed a significant interaction of Condition and Block [*F*_(1, 15)_ = 4.59, *p* < 0.05, η^2^*p* = 0.23], indicating that TPR was markedly increased in the random-reward condition but not in the contingent-reward condition, during the third block. A significant main effect of Condition in the HF component of HRV [*F*_(1, 15)_ = 5.63, *p* < 0.05, η^2^*p* = 0.27] was also observed, suggesting that parasympathetic activity was more enhanced in the random-reward condition compared with that in the contingent-reward condition. The LF/HF ratio of HRV showed no significant effects in either condition.

**Table 2:** **Means and standard errors of autonomic indices**.

	**Block**	**Contingent-reward**		**Random-reward**		**ANOVA**
		**Baseline**	**Task**	**Baseline**	**Task**	
MBP (mmHg)	1	79.08 (1.70)	83.26 (1.93)	76.12 (2.53)	79.62 (2.49)	C^*^, P^*^
	2	80.52 (1.85)	83.07 (1.46)	76.85 (2.08)	79.17 (2.10)	
	3	79.96 (1.83)	82.04 (1.72)	77.62 (1.92)	81.88 (1.65)	
HR (bpm)	1	65.06 (2.02)	67.16 (2.66)	63.77 (2.12)	64.95 (2.22)	C^*^
	2	65.84 (2.24)	66.54 (2.45)	63.50 (2.13)	64.15 (2.13)	
	3	65.24 (2.04)	66.78 (2.43)	63.98 (2.12)	65.47 (2.15)	
TPR (mmHg/l/min)	1	0.78 (0.05)	0.79 (0.06)	0.73 (0.05)	0.79 (0.06)	C × B^*^
	2	0.78 (0.05)	0.79 (0.05)	0.75 (0.05)	0.76 (0.05)	
	3	0.78 (0.05)	0.78 (0.05)	0.76 (0.06)	0.82 (0.06)	
LF/HF (ratio)	1	0.41 (0.10)	0.44 (0.08)	0.42 (0.09)	0.48 (0.09)	ns
	2	0.40 (0.09)	0.40 (0.12)	0.41 (0.09)	0.45 (0.09)	
	3	0.42 (0.10)	0.41 (0.10)	0.40 (0.13)	0.43 (0.11)	
HF (%)	1	51.16 (4.03)	55.53 (3.30)	53.07 (3.52)	61.70 (3.64)	C^*^
	2	50.09 (4.63)	50.09 (4.63)	51.15 (3.55)	57.54 (3.86)	
	3	52.31 (4.20)	52.05 (3.85)	51.14 (5.50)	55.32 (4.61)	
Epinephrine (pg/ml)	1	36.13 (18.18)	49.00 (33.44)	36.50 (34.93)	43.81 (45.72)	C^*^
	2	39.88 (25.30)	52.19 (57.64)	41.81 (45.47)	48.00 (49.81)	
	3	42.88 (36.04)	46.44 (35.79)	40.69 (38.69)	48.38 (44.41)	
Norepinephrine (pg/ml)	1	211.50 (71.50)	219.75 (84.51)	210.82 (58.97)	199.00 (83.15)	C × P^*^
	2	213.00 (69.96)	201.13 (69.90)	203.75 (59.25)	193.56 (63.36)	
	3	196.38 (63.00)	209.13 (72.78)	205.19 (58.29)	193.75 (54.19)	

MBP, mean blood pressure; HR, heart rate; TPR, total peripheral resistance; LF, low-frequency component of heart rate variability; HF, high-frequency component of heart rate variability. The column of ANOVA indicates significance of main effects of Condition (C), Block (B), and Period (P) and interactions (C × B, C × P, B × P, C × P × B) in analyses of variance;

* *p < 0.05; ns, non-significant*.

For catecholamine, epinephrine showed a significant main effect of Condition [*F*_(1, 15)_ = 6.10, *p* < 0.05, η^2^*p* = 0.29], indicating that overall concentration of epinephrine was higher in the contingent-reward condition compared with that in the random-reward condition. For norepinephrine, a significant interaction of Condition and Period was observed [*F*_(1, 15)_ = 5.55, *p* < 0.05, η^2^*p* = 0.27]. Further it was indicated that norepinephrine concentration did not change between baseline and task periods in the contingent-reward condition, while it was reduced during the task period in the random-reward condition.

### Associations of autonomic activity and decision-making

Table 3 shows the correlations within behavioral and autonomic indices in both conditions. In the contingent-reward condition, entropy was positively correlated with changes of norepinephrine, while response bias and reward acquisition were positively correlated with the HF component of HRV. MBP and TPR were positively correlated, suggesting sympathetic activity. The HF component of HRV and the LF/HF ratio of HRV were negatively correlated, suggesting that these parasympathetic and sympathetic indices worked in opposition to each other. Conversely, in the random-reward condition, no significant relations were found between autonomic indices and behavioral indices. In this condition, HR was correlated positively with norepinephrine and negatively with TPR. The HF component of HRV and the LF/HF ratio of HRV were also negatively correlated in this condition.

**Table 3:** **Correlations among behavioral and autonomic indices**.

**CONTINGENT-REWARD**										
	**Bias**	**Reward**	**Entropy**	**MBP**	**HR**	**TPR**	**LH/HF**	**HF**	**E**	**NE**
Bias	–	−0.77^*^	−0.02	0.27	0.19	0.22	−0.23	0.59^*^	0.00	−0.10
Reward		–	0.18	−0.05	0.38	−0.22	−0.22	0.51^*^	0.25	0.09
Entropy			–	−0.08	0.03	0.04	−0.31	0.21	0.33	0.63^*^
MBP				–	0.12	0.82^**^	−0.06	0.11	0.11	0.04
HR					–	−0.34	0.12	−0.04	0.72^*^	0.03
TPR						–	−0.58^*^	0.23	−0.17	−0.08
LH/HF							–	−0.58^*^	−0.23	0.07
HF								–	0.14	0.23
E									–	0.41
NE										–
**RANDOM-REWARD**										
Bias	–	0.18	0.06	−0.07	0.04	0.10	0.46	−0.40	0.08	0.16
Reward		–	0.15	−0.09	0.48	−30	−0.06	−0.03	0.11	0.34
Entropy			–	0.06	0.44	−0.17	−0.06	−0.01	0.33	0.22
MBP				–	0.18	0.16	0.25	−0.29	0.06	0.34
HR					–	−0.73^*^	0.26	−0.41	0.39	0.71^*^
TPR						–	−0.09	0.20	−0.32	−0.42
LH/HF							–	−0.96^**^	−0.18	0.20
HF								–	0.13	−0.23
E									–	0.41
NE										–

Bias, response bias; Reward, reward acquisition; MBP, mean blood pressure; HR, heart rate; TPR, total peripheral resistance; LH/HF, ratio of low-frequency and high-frequency components of heart rate variability; HF, high-frequency component of heart rate variability, E, epinephrine; NE, norepinephrine;

* p < 0.05;

** *p < 0.01*.

**Figure 2:**
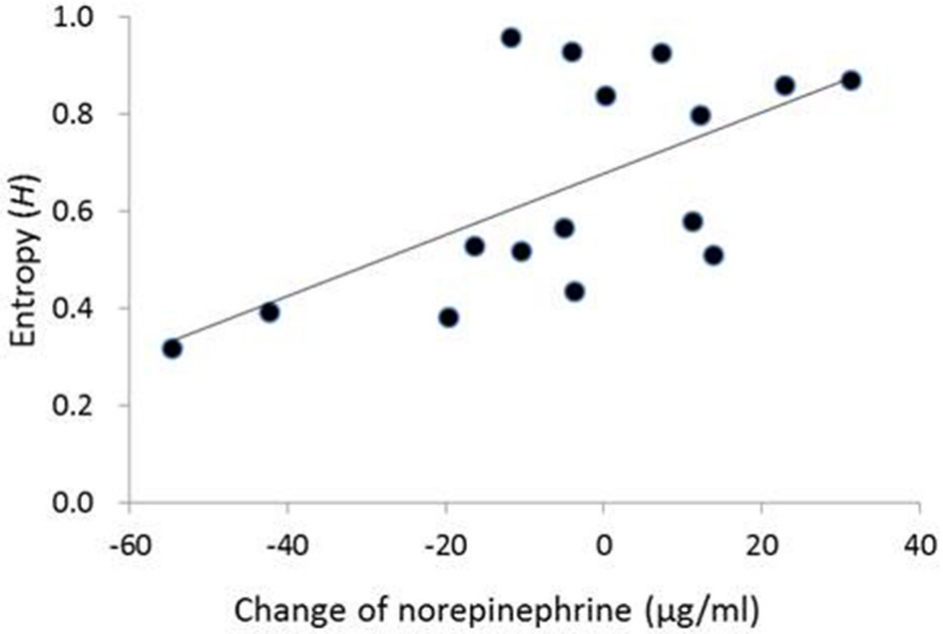
**Correlation between change of norepinephrine and entropy in decision-making in the contingent-reward condition**. No correlation between change of norepinephrine and entropy was observed in the random-reward condition. The vertical axis of the graph represents change of norepinephrine between before and after blocks of the task (i.e., positive/negative values mean increase/decrease of norepinephrine from the baseline in each block).

In the contingent-reward condition, a hierarchical regression analysis on entropy adopted a significant model [adjusted *R*^2^ = 0.44, *F*_(2,13)_ = 6.94, *p* < 0.01], including norepinephrine and the LF/HF ratio of HRV as independent variables. The analysis also revealed that the change of norepinephrine as an index of sympathetic activity (β = 0.65, *p* < 0.05), but not the LF/HF ratio of HRV, significantly and positively contributed to entropy (Figure 2). Conversely, in the random-reward condition, the regression model was not significant [*F*_(7,8)_ = 0.53, ns.].

### PET data

**Figure 3:**
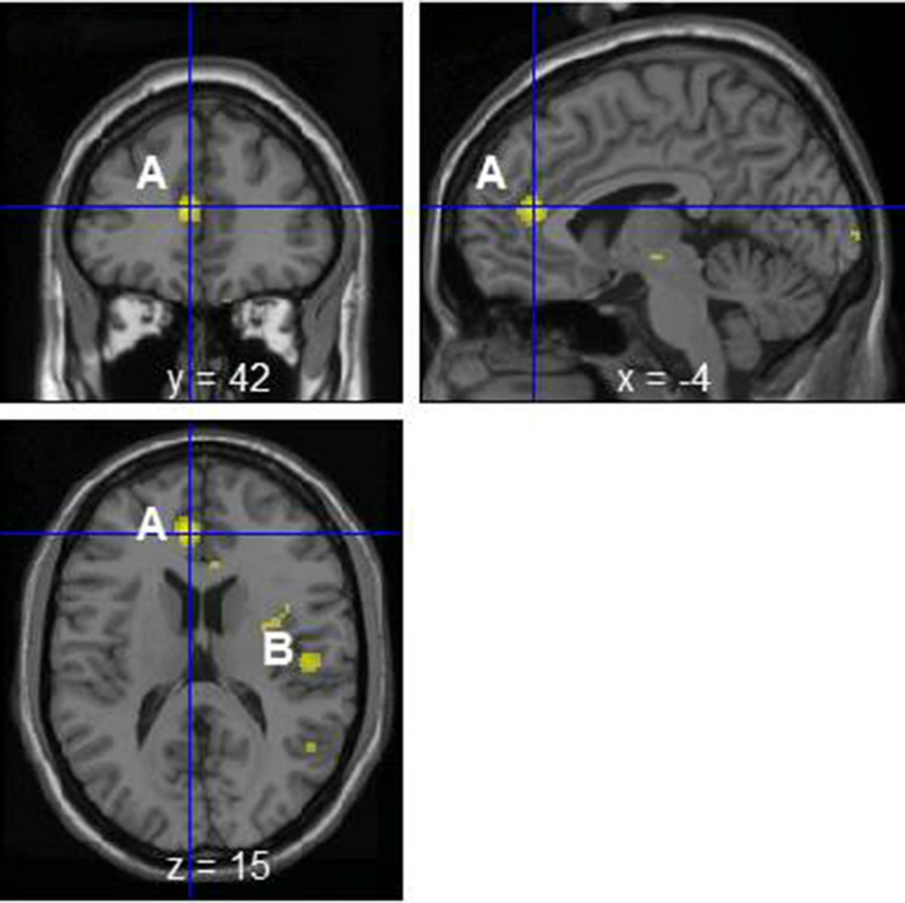
**Significant negative correlations between regional cerebral blood flow and change of norepinephrine in the contingent-reward condition**. A, Rostral anterior cingulate cortex; B, posterior insula.

The change of norepinephrine showed significant negative correlations with rCBF in brain regions including the parahippocampal gyrus, cerebellum, rostral ACC, right posterior insula, prefrontal cortex, globus pallidus, thalamus, putamen, and postcentral gyrus in the contingent-reward condition (Figure 3 and Table 4), while rCBF in the random-reward condition showed no significant correlations with norepinephrine in the frontal, limbic, and striatum regions. As already reported (Ohira et al., 2010), the HF component of HRV as an index of cardiovagal inhibitory control was positively correlated with rCBF in the rostral ACC and right DLPFC in the random-reward condition, but not in the contingent-reward condition. Other autonomic indices showed no significant correlations in either condition.

**Table 4:** **Significant negative correlations between rCBF and norepinephrine in contingent-reward condition**.

**Region**	**Side**	**BA**	***x***	***y***	***z***	**Z score**
Parahippocampal gyrus	L	28	−24	−6	−30	5.16
Cerebellum	R		14	−50	−28	4.35
Rostral anterior cingulate cortex	L	32	−6	42	16	4.33
Posterior insula	R		50	−16	18	3.88
Dorsal prefrontal cortex	L	8	−12	28	62	3.88
Globus pallidus	L		−14	−4	−2	3.83
Thalamus	R		5	−5	0	3.68
Putamen	R		24	8	−2	3.66
Cerebellum	L		−22	−40	−18	3.48
Postcentral gyrus	R	2	50	−28	40	3.19

*Coordinates are in MNI space (SPM99). R, right; L, left; BA, Brodmann's area; x, y, z, three-dimensional coordinates used to determine a voxel referring to medial-lateral (x: positive = right), anterior-posterior (y: positive = anterior), and superior-inferior (z: positive = superior) positions; rCBF, regional cerebral blood flow*.

**Figure 4:**
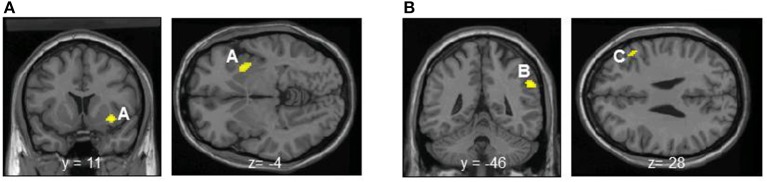
**(A)** Significant negative correlations between regional cerebral blood flow and entropy in decision-making in the contingent-reward condition. A, Anterior insula. **(B)** Significant positive correlations between regional cerebral blood flow and entropy in decision-making in the random-reward condition. B, Inferior parietal lobule; C, dorsolateral prefrontal cortex.

**Figure 5:**
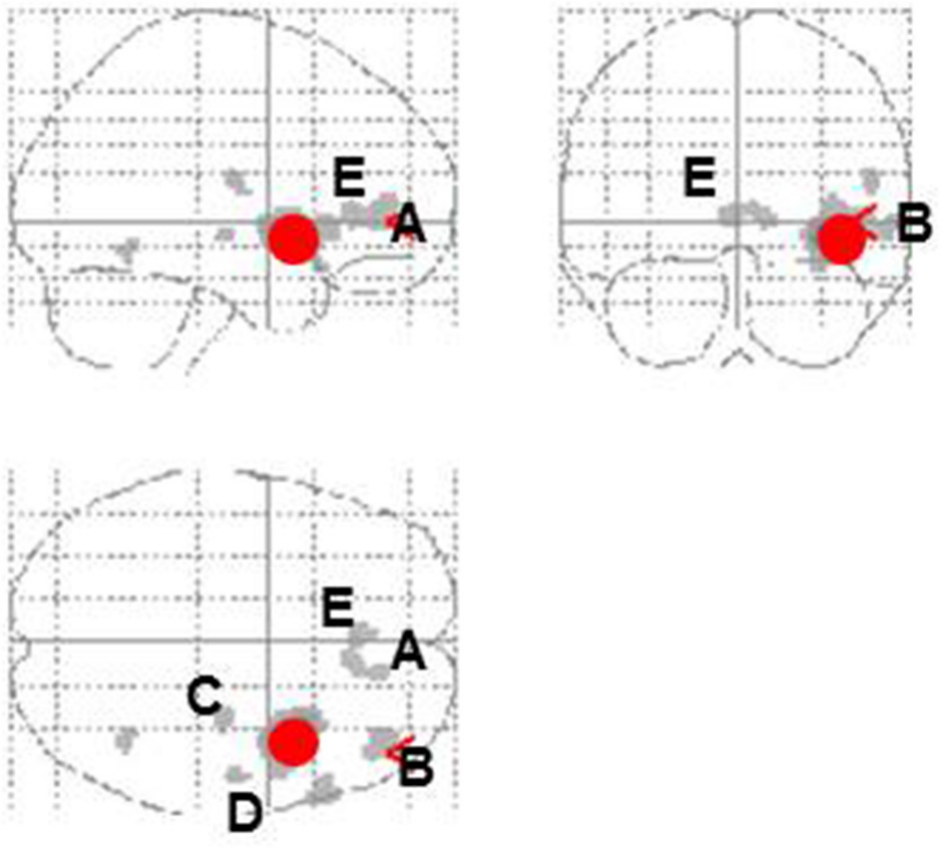
**Correlational activity between the right insula (red circle) and other brain regions in the contingent-reward condition**. A, Ventromedial prefrontal cortex; B, lateral prefrontal cortex; C, putamen; D, posterior insula; E, rostral anterior cingulate cortex.

Entropy showed significant negative correlations with rCBF in the right anterior insula and superior temporal gyrus in the contingent-reward condition (Figure 4A and Table 5). In the random-reward condition, entropy was positively correlated with rCBF in the right inferior parietal lobule and bilateral DLPFC (Figure 4B and Table 5). Functional connectivity between brain regions that were related to exploration in the contingent-reward condition was examined by a further correlation analysis of the whole-brain using rCBF values from a cluster indicating the highest correlation with entropy (the right anterior insula) as a seed. As a result, activity in the right anterior insula was positively correlated with activity in several region in the right hemisphere including the ventrolateral prefrontal cortex (VLPFC: BA47, *x* = 60, *y* = 22, *z* = −4, *Z* = 4.11), lateral prefrontal cortex (LPFC: BA46, *x* = 38, *y* = 48, *z* = 8, *Z* = 4.08), putamen (*x* = 30, *y* = −18, *z* = −4, *Z* = 3.98), posterior insula (*x* = 32, *y* = −12, *z* = 18, *Z* = 3.81), and rostral ACC (*x* = 8, *y* = 32, *z* = 4, *Z* = 3.56) (Figure 5).

**Table 5:** **Significant negative correlations between rCBF and entropy in the contingent-reward condition and positive correlations between rCBF and entropy in the random-reward condition**.

**Region**	**Side**	**BA**	***x***	***y***	***z***	**Z score**
**CONTINGENT-REWARD**						
Anterior insula	R		34	12	−6	4.29
Superior temporal gyrus	R	22	58	0	4	3.29
**RANDOM-REWARD**						
Inferior parietal lobule	R	40	58	−46	34	3.81
Dorsolateral prefrontal cortex	R	46	48	28	44	3.42
Dorsolateral prefrontal cortex	R	8	54	20	28	3.32
Dorsolateral prefrontal cortex	L	46	−38	28	34	3.23

*Coordinates are in MNI space (SPM99). R, right; L, left; BA, Brodmann's area; x, y, z, three-dimensional coordinates used to determine a voxel referring to medial-lateral (x: positive = right), anterior-posterior (y: positive = anterior), and superior-inferior (z: positive = superior) positions; rCBF, regional cerebral blood flow*.

Correlations between sympathetic activity, rCBF, and entropy described above were examined in each condition, separately. Due to the small sample size, formal statistical tests did not show any significant differences of the correlations between the contingent-reward condition and the random-reward condition. Therefore, results of the present study must be interpreted with a caution.

## Discussion

As predicted, sympathetic activity indexed by changes of norepinephrine was linked with exploration in decision-making represented by entropy. Activity of brain regions including the insula was associated with the correlation between sympathetic activity and exploration, in the contingent-reward condition where an appropriate option was stochastically determined and thus uncertainty in decision-making was relatively low. However, in the random-reward condition where uncertainty in decision-making was extremely high, exploration in decision-making was not linked with sympathetic activity but with brain activity in the DLPFC and inferior parietal lobule. These findings suggest that the linkage between sympathetic activity and decision-making might be, at least partly, dependent on the degree of uncertainty of a situation. Probabilities of response bias and reward acquisition were matched to the contingencies between options and outcomes both in the contingent-reward condition and in the random-reward condition (approximately 70 and 50%, respectively, see Table 1), suggesting validity of experimental manipulation in this study. Values of entropy in the two conditions of this study were consistent with those in our previous study where a similar decision-making task was used (Ohira et al., 2013), suggesting reliability of this index of exploration.

Only catecholamine but no other indices of sympathetic activity (the LF/HF component of HRV, MBP, HR, and TPR) predicted entropy in the contingent-reward condition. This seemed that signals of peripheral sympathetic activity affecting exploration are conveyed to the brain mainly via the neurochemical route including the afferent vagus nerve expressing β-adrenergic receptors, NTS, LC-norepinephrine system, and basal forebrain cholinergic system, as proposed by several researchers (e.g., Williams and McGaugh, 1993; Cahill and McGaugh, 1998; Clayton and Williams, 2000; Cahill and Alkire, 2003; Berntson et al., 2003, 2011), while the somatosensory signals driven by cardiovascular responses might play relatively minor roles in modulation of exploration. In addition, catecholamine and other sympathetic indices did not affect response bias or reward acquisition, suggesting that sympathetic activity is associated with exploration in decision-making, but not with currently appropriate strategies (exploitation).

Neither catecholamine nor cardiovascular indices were associated with entropy in the random-reward condition, where sympathetic activity was generally attenuated. This attenuation of sympathetic activity in such a highly uncertain condition has been reported in our previous studies (Kimura et al., 2007; Ohira et al., 2009, 2010), and corresponds to a typical physiological coping style to a stressful situation that is difficult to control and individuals experience insufficient resources (Blascovich et al., 1999; Keay and Bandler, 2001). Attenuation of cardiovascular activity in the random-reward condition suggests prevention of energy expenditure by reduction of allocation of energy to ongoing behaviors that have become inappropriate, and allocation of the saved energy to attention and cognitive processes to find a way to more appropriate coping. This result suggests that autonomic responses accompanying decision-making should be under the top-down regulation on the basis of appraisal for the current situation (Ohira et al., 2010; Studer and Clark, 2011; Stankovic et al., 2014). It should be noted that the average value of entropy was maintained at a high level in the random-reward condition (see Table 1), suggesting that participants did not abandon efforts for the task and did not just adopt simple strategies of decision-making (e.g., choice of the same option in all trials), even in the random-reward condition.

Changes of norepinephrine but not those of epinephrine specifically correlated with entropy in the contingent-reward condition of the present study, while epinephrine but not norepinephrine correlated with entropy in our previous study (Ohira et al., 2013). Although reasons for this difference are not clear, it is possible that the correlation between changes of norepinephrine and entropy in the contingent-reward condition of the present study was produced mainly by a decrease in norepinephrine level. Figure 2 showed that a decrease of norepinephrine from baseline (under the “0” level) was associated with lower values of entropy. These results suggest that some participants showed a reduction of sympathetic activity that accompanied the progress and establishment of learning about the contingency between options and outcomes. A decrease of norepinephrine might sensitively reflect such a reduction of sympathetic activity, while the concentrations of epinephrine in this study were maintained at high levels (see Table 2). Norepinephrine is the primary transmitter in the sympathetic nerve, while epinephrine is a secondary product that is synthesized and secreted in the adrenal medulla. Thus, norepinephrine might have higher temporal reactivity than epinephrine because levels of norepinephrine (but not epinephrine) are mainly modulated by the norepinephrine transporter that enables rapid shut-out of responses (Schroeder and Jordan, 2012). Furthermore, the rate in metabolism is higher for norepinephrine compared to epinephrine (Eisenhofer and Finberg, 1994).

The changes of norepinephrine in the contingent-reward condition were negatively correlated with rCBF in brain regions including the right insula and ACC, as well as the limbic and striatum regions such as the parahippocampal gyrus, thalamus, globus pallidus, and putamen, which have tight connections with the insula (Augustine, 1996). Neural activity in these brain regions related to bodily responses such as skin conductance responses (Critchley et al., 2002), inflammation induced by vaccination (Harrison et al., 2009), interoceptive awareness (Pollatos et al., 2007), and the increase of epinephrine in reversal learning (Ohira et al., 2013), has been repeatedly reported. In addition, the brain regions in which activity showed correlations with norepinephrine changes in the present study are included in the neural network whose functional connectivity in a resting-state showed synchronization with skin conductance as an index of sympathetic activity (Fan et al., 2012). As the insula and ACC are the top-level centers of the ascending pathways of information flow from the body to the brain including changes of catecholamine, mainly via the afferent vagus nerve, brain norepinephrine system, and basal forebrain cholinergic system (Berntson et al., 2003, 2011), our data provide additional evidence for the role of the insula and ACC to produce neural representations of bodily states (Craig, 2009; Critchley, 2009).

Nevertheless, the negative correlation between changes of norepinephrine and rCBF in the insula and the ACC observed in the present study seems to contradict our previous finding that changes of epinephrine were positively correlated with rCBF in those brain regions (Ohira et al., 2013). This discrepancy can be interpreted by considering that the insula does not respond just linearly to inputs of peripheral bodily signals, but might work as a “comparator.” Seth (2013) and their colleagues (Seth et al., 2012) argued that the insula can detect a mismatch (prediction error) between predicted bodily responses calculated by an inner model and actual inputs of bodily responses. The greater the mismatch between predicted bodily responses and actual bodily responses is, the larger insular activity should happen. The findings of the present study and our previous study (Ohira et al., 2013) seem consistent with this notion; specifically, we speculate that the insula and the connected neural network detected a positive prediction error (the increase of bodily responses compared to the current adaptation level) in the previous study and detected a negative prediction error (the decrease of bodily responses compared to the current adaptation level) of bodily responses in the present study. The negative correlation between the explorative tendency in decision-making indexed by entropy and rCBF in regions including the insula also seems to support this concept. Namely, activity of the “comparator” neural network including the anterior and posterior portions of the insula, driven by detection of the decrease of sympathetic activity compared to the current adaptation level, might lead to reduction of the explorative tendency in decision-making. The positive correlations between activity in the right anterior insula and other brain regions such as the VLPFC, LPFC, rostral ACC, and striatum suggest that the prediction error detected in the insula might serve to modulate activity in the frontal-striatum neural network that is directly involved in decision-making (e.g., van Leijenhorst et al., 2006; Eshel et al., 2007; Costa and Averbeck, 2013). Additionally the detected prediction error can be utilized to tune the strategy of decision-making in the dimension of exploration and exploitation (e.g., Daw et al., 2006; Frank et al., 2009; Sallet and Rushworth, 2009).

Exploration in decision-making indexed by entropy was positively correlated with rCBF in the bilateral DLPFC and inferior parietal lobule, but not with rCBF in the insula, in the random-reward condition. This result is consistent with previous findings showing that the prefrontal and parietal neural network is involved in exploration in several decision-making tasks (Daw et al., 2006; Sallet and Rushworth, 2009; Costa and Averbeck, 2013). We previously reported higher activation of the DLPFC in the random-reward condition than in the contingent-reward condition (Ohira et al., 2010). The DLPFC is involved in working memory, executive control, and top-down control over flow of information processing (Seo et al., 2007). Thus, the DLPFC might be more recruited during decision-making in a highly uncertain situation where continuous seeking for hidden rules on the basis of memorizing past experiences of own actions and the outcomes is required. Such cognitive functions may lead to exploratory seeking for an appropriate strategy of decision-making in the uncertain situation. Furthermore, the right DLPFC plays a critical role in the inhibitory control of superficially seductive options (Fecteau et al., 2007). This function likely contributes to exploration by inhibition of simple sticking to just recent gains. A neuroimaging study using ^15^O-PET showed that the left side of the DLPFC is critical for generation of randomness of behavioral sequences (Jahanshahi et al., 2000), and the causality of this notion was verified in a study using transcranial magnetic stimulation (Jahanshahi and Dirnberger, 1999). This function may also support exploration by avoiding simple behavioral patterns such as thoughtless repeats of previous choices or the Win-stay Lose-shift strategy. In addition, it has been suggested that the inferior parietal lobule works as an interface of frontal areas where values of options are calculated and motor output is controlled (Daw et al., 2006). Activity in such a frontal-parietal network was also shown to correlate with the amount of information that participants gathered before committing to a decision (Furl and Averbeck, 2011). In contrast to the findings in the contingent-reward condition, there were no correlations between sympathetic indices including norepinephrine with exploration in decision-making or activity of brain regions including the insula in the random-reward condition. The positive correlation between the HF component of HRV as an index of cardiovagal activity and rCBF in the rostral ACC and right DLPFC in this condition suggests that physiological responses are under inhibitory control on the basis of evaluation of the current situation in the frontal neural network (Thayer et al., 2012). Such neural processes likely canceled the effects of sympathetic activity on exploration in the random-reward condition.

It has been well known that activity of dopamine neurons in the midbrain-striatum neural circuit is the largest when uncertainty of delivery of reward is the highest (Fiorillo et al., 2003). This classical finding is consistent with the result of our previous study (Ohira et al., 2010) showing that activation of the dorsal striatum, which is a main target area of projection of midbrain dopamine neurons, was higher in the random-reward condition (higher uncertainty) than in the contingent-reward condition (lower uncertainty). On the other hand, entropy showed no correlation with activation of the midbrain-striatum dopamine circuit in both conditions in this study. Taken together, while activity in dopamine neurons might involve coding and evaluation of uncertainty in decision-making, the neural networks including the insula and DLPFC might involve modulation of exploration in decision-making on the basis of such coding and evaluation of uncertainty.

Some limitations of the present study should be noticed. First, as the sample size of this study was small and participants were all male, the generalizability of findings of this study should be further examined. Secondly, neuroimaging using PET has limited temporal resolution compared to fMRI. Also, as PET studies are largely correlative and we used relatively liberal statistical standards, the causality of these findings should be interpreted cautiously. Thirdly, the decision-making task used in the present study was minimally simple one with only two alternative options. Tasks with multiple alternative options like the task used in the study by Daw et al. (2006) might be more useful to draw dynamic characteristics of exploration in detail. Nevertheless, we replicated our previous finding that sympathetic activity correlates with exploration in decision-making indexed by entropy, and that this association between sympathetic activity and exploration can be at least partly mediated by insular activity. We also expanded this notion by showing that functions of such a brain-body circuit affecting exploration can vary according to the degree of uncertainty of a situation in decision-making. As a source of inconsistency of the relationship between sympathetic activity and decision-making (Dunn et al., 2006; Rolls, 2014), it has been shown that individual differences in sensitivity to one's own sympathetic activity (interoception) can moderate the relationship (Sokol-Hessner et al., 2014; Wölk et al., 2014). The present study suggested that uncertainty of the situation of decision-making might also be an additional moderator of the relationship.

## Author contributions

Hideki Ohira, Naho Ichikawa, and Kenta Kimura contributed to study design. Naho Ichikawa and Kenta Kimura contributed to measurements and analyses of behavioral and autonomic data. Seisuke Fukuyama was responsible for data-acquisition in neuroimaging using PET with the supervision of Jun Shinoda and Jitsuhiro Yamada. Hideki Ohira interpreted the data with helps of Naho Ichikawa and Kenta Kimura, and wrote the manuscript. All authors approved the final version of the paper.

## Funding

This study was supported by a Grant-in-Aid for Scientific Research of the Japan Society for the Promotion of Science (No. 16330136) and a Grant-in-Aid for Scientific Research on Innovative Areas (Research in a Proposed Research Area) 2010 (No. 4102-21119003) from the Ministry of Education, Culture, Sports, Science and Technology, Japan.

### Conflict of interest statement

The authors declare that the research was conducted in the absence of any commercial or financial relationships that could be construed as a potential conflict of interest.
